# Persisting social disadvantage and intercensal social mobility in Northern Ireland: an examination of morbidity and all-cause mortality using three waves of the Northern Ireland Longitudinal Study (NILS).

**DOI:** 10.23889/ijpds.v9i5.1662

**Published:** 2024-09-18

**Authors:** Rachel McCarter, Michael Rosato, Gerard Leavey

**Affiliations:** 1 Bamford Centre for Mental Health and Wellbeing, Ulster University; 2 Administrative Data Research – Northern Ireland (ADR-NI), Ulster University

## Objective and Approach

Using census returns from three waves of NILS (1991, 2001, 2011) linked with mortality data, we examined morbidity/mortality outcomes by socio-economic disadvantage over time (using an individual-level deprivation index derived from educational attainment, social class, housing tenure and household car availability at each census). These were combined to generate two indicators of Intercensal social mobility: measuring no change, and upward/downward mobility between censuses. The relationship between mobility (2001-2011) and (separately) self-reported mental ill-health (MIH) at the 2011 Census and all-cause mortality (2011-2015) were examined using logistic regression and Cox PH modelling respectively.

## Results

Population comprised 288,262 individuals aged 25-74 in 2011, recording 19,318 deaths (2011-2015) and 23,959 (8.3%) reporting MIH. MIH: those with no intercensal mobility followed the standard pathway associated with persisting disadvantage (comparing with the most advantaged the least advantaged recorded OR=16.13:95%CI=13.50,19.28); and while both the upwardly and downwardly mobile generally recorded higher ORs than those consistently most advantaged, the magnitude of ORs increased with social distance traversed; and ORs for downward mobility were consistently higher than with upward mobility. Patterns associated with all-cause mortality were similar.

## Conclusions

This suggests: upward mobility retains something of the social patterning pertaining in social disadvantage levels left behind; downward mobility may be connected to ongoing health issues. Poor health outcomes remain strongly associated with socio-economic circumstance.

## Implications

Research focusing on relationship between social mobility, disadvantage and health outcomes in NI is limited: access to more precise administrative data will enhance possibilities associated with evidence-based policy formation.

